# Myasthenic crisis and late deep vein thrombosis following thymectomy in a patient with myasthenia gravis: A case report

**DOI:** 10.1097/MD.0000000000019781

**Published:** 2020-04-10

**Authors:** Cheng-Yuan Lin, Wei-Cheng Liu, Min-Hsien Chiang, I-Ting Tsai, Jen-Yin Chen, Wan-Jung Cheng, Chun-Ning Ho, Shu-Wei Liao, Chin-Chen Chu, Cheuk-Kwan Sun, Kuo-Chuan Hung

**Affiliations:** a Department of Anesthesiology, Chi Mei Medical Center, Tainan; b Department of Anesthesiology, Kaohsiung Chang Gung Memorial Hospital and Chang Gung University College of Medicine; c Department of Emergency Medicine, E-Da Hospital; d College of Medicine, I-Shou University, Kaohsiung,; e Department of the Senior Citizen Service Management, Chia Nan University of Pharmacy and Science, Tainan, Taiwan.

**Keywords:** deep vein thrombosis, hyperlactatemia, myasthenia gravis, myasthenic crisis, thymectomy

## Abstract

**Introduction:**

Surgical stress and pain are potential provoking factors for postoperative myasthenic crisis (POMC). We report the occurrence of early POMC and late deep vein thrombosis (DVT) in a man with myasthenia gravis (MG) undergoing thymectomy, addressing possible link between reversal of opioid overdose with naloxone and the triggering of POMC.

**Patient concerns:**

A 71-year-old man with impaired renal function (ie, estimated glomerular filtration rate [egfr]: 49.1 mL/min/1.73 m^2^) with diagnosis of MG made 2 months ago was scheduled for thymectomy. After uncomplicated surgery, he experienced opioid overdose that was treated with naloxone. Hyperlactatemia then developed with a concomitant episode of hypertension. Three hours after reversal, he suffered from myasthenic crisis presenting with respiratory failure and difficult weaning from mechanical ventilation.

**Diagnosis:**

Stress-induced hyperlactatemia and subsequent myasthenic crisis

**Interventions:**

Pyridostigmine and immunosuppressive therapy with prednisolone were initiated. Hyperlactatemia subsided on postoperative day (POD) 5. Tracheal extubation was performed successfully on POD 6.

**Outcomes:**

During the course of hospitalization, his eGFR (ie, 88.9 mL/min/1.73 m^2^) was found to improve postoperatively. After discharge from hospital, he developed DVT in the left femoral and popliteal veins on POD 24 when he was readmitted for immediate treatment with low-molecular-weight heparin. He was discharged without sequelae on POD 31. There was no recurrence of myasthenic crisis or DVT at 3-month follow-up.

**Conclusions:**

Following naloxone administration, hyperlactatemia may be an indicator of pain-related stress response, which is a potential provoking factor for myasthenic crisis. Additionally, patients with MG may have an increased risk of DVT possibly attributable to immune-mediated inflammation. These findings highlight the importance of perioperative avoidance of provoking factors including monitoring of stress-induced elevations in serum lactate concentration, close postoperative surveying for myasthenic crisis, and early recognition of possible thromboembolic complications in this patient population.

## 1 Introduction

Myasthenia gravis (MG), an autoimmune antibody-mediated disease that affects the neuromuscular junction, is characterized by fluctuating weakness of voluntary muscles, in particular the extraocular, bulbar, and proximal limb muscles.^[1]^ It is considered to be a rare disease with an overall incidence rate of about 0.01 per 1,000 persons/yr in the United States.^[1]^ Although thymectomy is the first-line therapy for thymomatous MG patients,^2^,^3^ various medications, surgical stress, and anesthetic agents may trigger postoperative myasthenic crisis (POMC) after this procedure.^4^,^5^ We reported the occurrence of POMC in a man who developed hyperlactatemia after naloxone administration for opioid overdose. During the course of hospitalization, his renal function was found to improve after thymectomy. He also developed late deep vein thrombosis (DVT) after discharge from hospital. The associations among MG, DVT, and renal pathology as well as the possible link between perioperative hyperlactatemia and POMC were also discussed. Written consent was obtained from the patient.

## 2 Case presentation

**Figure 1:**
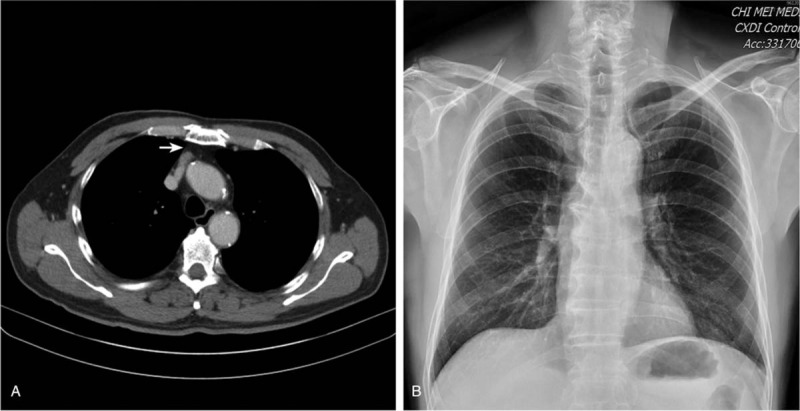
(A) Thymic hyperplasia on thoracic computed tomography (CT) (arrow); (B) Unremarkable finding on preoperative chest radiograph, indicating unlikely non-pulmonary origin of postoperative respiratory distress. CT = computed tomography.

A 71-year-old man, non-smoker (height: 155 cm; weight: 59 kg), was scheduled to receive video-assisted thoracoscopic extended thymectomy with the diagnosis of MG. Two months previously, he developed symptoms of right ptosis and progressive swallowing difficulty. Based on a positive response to edrophonium and increased titers of autoantibodies to acetylcholine receptor (19.3 nmol/L; normal < 0.2 nmol/L), he was diagnosed as having MG with severity belonging to Osserman's classification IIb (ie, generalized moderate weakness and/or bulbar dysfunction).^[6]^ Thoracic computed tomography demonstrated glandular hyperplasia of the thymus (Fig. 1A). The patient was started on prednisolone 20 mg daily and pyridostigmine 60 mg three times daily. His past history included hypertension without evidence of previous myasthenic crisis or thromboembolic events (eg, history of lower limb swelling). The results of electrocardiography, pulmonary function test [eg, vital capacity: 93%], echocardiography (eg, left ventricular ejection fraction: 85.1%), chest radiography (Fig. 1B), and laboratory studies (eg, coagulation test) were unremarkable. On the other hand, impaired renal function [i.e., serum creatinine: 1.42 mg/dL; eGFR: 49.1 mL/min/1.73 m^2^] was observed after admission.

Preoperative physical examination of the patient showed clear consciousness without respiratory distress. Vital signs included a blood pressure of 187/103 mm Hg, heart rate of 82 beats/min, and respiratory rate of 14 breaths/minute. Under real-time neuromuscular monitoring with a train-of-four (TOF) monitor (TOF-watch SX, N.V. Organon, Oss, Netherlands), anesthesia was induced with propofol (130 mg) and rocuronium (0.85 mg/kg). Following successful tracheal intubation with a double-lumen tracheal tube (Broncho-Cath; Mallinckrodt, Athlone, Ireland), general anesthesia was maintained with sevoflurane, rocuronium (total dosage: 40 mg), and a continuous infusion of remifentanil. An 18-gauge peripheral intravenous line and an arterial line were introduced. The surgical time was 4 hours 15 minutes with an estimated blood loss of 100 mL. Upon completion of surgery, sugammadex 4 mg/kg was administered to reverse neuromuscular blockade, with a maximum TOF ratio of 0.93 following reversal. Additionally, intravenous morphine 8 mg was given for postoperative analgesia. After successful extubation in the operating room and resumption of spontaneous breathing, he was transferred to the post-anesthesia care unit (PACU) for further care.

**Figure 2:**
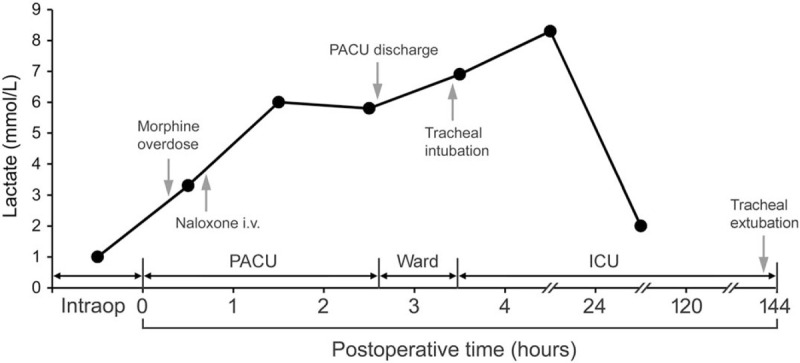
Change in serum lactate concentration during perioperative period with important events marked on the timeline. ICU = intensive care unit, Intraop = intraoperative period, PACU = post-anesthesia care unit.

During the immediate postoperative period, the patient was hemodynamically stable without respiratory distress. Because of surgical pain with a numeric rating scale of 5 (scale of 0–10), intravenous morphine was titrated to a total dosage of 7 mg. Forty-five minutes later, respiratory distress with drowsiness was noted. Physical examination found pinpoint pupils with a TOF ratio of 0.9. Blood gas analysis demonstrated severe hypercapnia (arterial carbon dioxide pressure: 117.7 mm Hg) and acidosis (pH: 6.996, lactate levels: 3.3 mmol/L). On suspicion of morphine overdose, intravenous naloxone was administered twice (0.08 mg each time). After 20 minutes, the patient regained consciousness and normal respiratory pattern. Subsequent blood gas analysis demonstrated hyperlactatemia (lactate levels: 6.0 mmol/L) (Fig. 2) despite improvement in arterial carbon dioxide pressure and arterial oxygen pressure after naloxone administration. Taking into account the overall clinical improvement, he was transferred to ward with spontaneous breathing and stable hemodynamics after observation for 100 minutes following initial reversal of opioid overdose.

However, one hour after being transferred to ward (ie, three hours after naloxone administration), he was found to exhibit consciousness loss, respiratory distress, and pinpoint pupils. Intravenous naloxone 0.4 mg was given, followed by endotracheal intubation and transfer to the intensive care unit for mechanical ventilatory support. Brain computed tomography showed no intracranial lesion. He regained consciousness two hours after naloxone administration in the intensive care unit without symptoms of opioid withdrawal (eg, pulmonary edema).^[7]^ As weaning from mechanical ventilation was difficult on POD 3, a diagnosis of POMC was made.^[1]^ Steroid therapy (prednisolone 40 mg twice daily) and pyridostigmine (60 mg 3 times a day) was initiated. The patient was extubated smoothly on POD 6 and was discharged from hospital on POD 12. The course of hyperlactatemia was shown in Figure 2. In addition, his eGFR increased from 49.1 mL/min/1.73 m^2^ at baseline to 88.9 ml/min/1.73m^2^ on POD 5. Pathological analysis of the specimen from thymectomy confirmed the diagnosis of type B2 thymoma according to the World Health Organization (WHO) classification.^[8]^

The patient was readmitted on POD 24 because of left thigh swelling. Ultrasonographic examination showed evidence of DVT involving the left femoral and popliteal veins. Anticoagulant therapy with low-molecular-weight heparin (ie, subcutaneous Clexane 60 mg every 12 hours) was implemented immediately after hospitalization, and he was discharged without sequelae on POD 31. During hospitalization, his eGFR was 86.5 mL/min/1.73m^2^. There was no recurrence of myasthenic crisis or DVT up to 3 months of follow-ups.

## 3 Discussion

Owing to the reduced number of functioning nicotinic acetylcholine receptors, even small amounts of nondepolarizing neuromuscular blocking agents can lead to profound neuromuscular blockade in patients with MG.^[9]^ Reversal of neuromuscular blockade in patients with MG by sugammadex has been reported to result in rapid and complete recovery of neuromuscular function without signs of postoperative residual neuromuscular blockade.^[9]^ In our patient with respiratory distress in the PACU, postoperative recurrence of neuromuscular blockade (ie, recurarization) was unlikely based on a TOF ratio of 0.9. The diagnosis of opioid overdose was made based on the typical clinical symptoms and signs (ie, pinpoint pupils, respiratory distress, drowsiness) as well as symptom improvement after naloxone administration. Since the serum half-life of naloxone is approximately 60 minutes,^[7]^ surgical patients receiving naloxone for reversal of opioid overdose is recommended to be observed for 90 minutes.^[7]^ Although our patient did not develop renarcosis in the PACU after observation for 100 minutes, it occurred about 3 hours after initial reversal. Patients with MG have been reported to demonstrate enhanced sensitivity to opioid,^4^,^10^ which may at least partially explain the delayed renarcosis after initial reversal in our patient.

MG is a disease of young women and old men with an overall mortality rate of 2.2%.^[1]^ Myasthenic crisis, which occurs in approximately 15% to 20% of MG patients within the first 2 years of the diagnosis^[11]^ with a mortality rate of 4% to 4.47%,^1^,^12^ is characterized by respiratory failure requiring invasive or noninvasive mechanical ventilation.^[1]^ Old age and respiratory failure requiring tracheal intubation were identified as predictors of mortality.^[1]^ Of all patients with MG undergoing thymectomy, 10% to 11.5% may experience POMC and require intubation with a median duration of intubation up to 9 days (range: 2–28 days).^13^,^14^ Several predisposing factors were identified for the occurrence of POMC, including preoperative bulbar symptoms, intraoperative blood loss >1000 mL, preoperative serum level of anti-acetylcholine receptor antibody >100 nmol/L,^[14]^ and WHO histologic classification B2–B3 thymoma.^[13]^ Accordingly, the risk factors for POMC in our patient included the presence of preoperative bulbar symptoms and WHO histologic thymoma grading of B2.

A previous study developed a clinical score for the prediction of POMC after taking into account MG-associated compromise of respiratory function, disease duration, and extent of muscle involvement.^[15]^ The clinical predictive score, which included vital capacity <80% (yes = 3, no = 0), disease duration <3 months (yes = 2, no = 0), and bulbar symptoms immediately before thymectomy (yes = 1, no = 0), yields a total score ranging from 0 to 6.^[15]^ The probability of postoperative crisis was 0.9% and 25.9% for low (less than 3) and high (3 or more) score groups, respectively. According to this scoring system, the risk score of our patient was 3, indicating a high risk of myasthenia crisis.

The occurrence of respiratory failure 3 hours after opioid reversal could be attributed to the development of concomitant renarcosis and of POMC in our patient. As stress (ie, surgery) and pain are known potential triggers for POMC,^5^,^10^ we titrated the lowest effective dose of naloxone (ie, total dosage of 0.16 mg) to minimize the risk of opioid withdrawal. Despite the precautions taken, reversal of morphine-associated analgesia may cause pain-induced stress. Interestingly, we found the development of hyperlactatemia, which subsided on POD 5 (lactate levels: 2 mmol/L), after initial reversal (Fig. 2). Previous studies have shown that hyperlactatemia can result from a stress response because stress-induced sympathetic nervous activation and increase in metabolic rate may contribute to accelerated glycolysis and an altered energy utilization.^16^,^17^ As there was no intraoperative hemodynamic instability to explain the possible development of ischemic hyperlactatemia, stress hyperlactatemia after initial reversal could be the diagnosis in our patient. Therefore, care should be taken when naloxone is used for opioid reversal in patients with MG undergoing thymectomy because the resulting stress, as reflected by an elevated serum lactate level, may trigger POMC. Nevertheless, the predictive value of hyperlactatemia for POMC in this patient population remains to be elucidated.

DVT occurred in our patient on POD 24. In general, stasis of blood flow, hypercoagulable state, and vascular endothelial cell damage are the major factors that predispose to DVT (ie, “Virchow's triad”).^[18]^ The risk factors for DVT have been reported to be setting-related (eg, major general surgery and trauma) and patient-related (eg, old age and malignancy).^19^,^20^ In our patient, the predisposing factors for DVT included old age and recent surgery.^19^,^20^ In addition, recent evidence suggests that immune-mediated diseases may play a role in DVT occurrence,^21^,^22^ probably due to inflammation-induced activation of proinflammatory cytokines and adhesion molecules as well as elevation of clotting factor levels that contribute to the development of venous thromboembolism.^23^,^24^ Indeed, a previous large-scale study demonstrated that there were increased risks of DVT in patients with immune-mediated diseases.^[21]^ In concert with this finding, a case study proposed that MG-related endothelial injury may be responsible for the development of DVT and the life-threatening pulmonary embolism.^[22]^ Our report may further support an increased risk of DVT in patients with immune-mediated diseases. Therefore, anticoagulation therapy and constant monitoring for venous thrombosis disease may be considered for patients with MG even after thymectomy because it may take up to 5 years postoperatively to achieve a stable remission of the symptoms and signs of MG.^[25]^ Considering the risk of thromboembolism, pulmonary embolism^[22]^ should be included on the list of differential diagnoses other than myasthenic crisis on encountering a patient with a known diagnosis of MG who presents with respiratory distress.

Another interesting finding in our patient was that his eGFR improved from 49.1 mL/min/1.73m^2^ at baseline to 88.9 mL/min/1.73m^2^ as soon as POD 5 and remained stable on POD 26 (86.5 mL/min/1.73 m^2^). Although the reasons remain unclear, MG is known to be associated with various immunological diseases including glomerulonephritis.^26^,^27^ Additionally, the presence of thymoma may also be related to minimal change nephrotic syndrome.^[28]^ An isolated case report also demonstrated improvement in renal function at a 4-month follow-up after thymectomy in a patient with concomitant MG and membranous nephropathy.^[29]^ The authors^[29]^ suggested a possible role of MG-mediated impairment of cellular immunity or the presence of anti- acetylcholine receptor autoantibodies antibodies in the pathogenesis of glomerulonephritis. Therefore, whether the prompt improvement in renal function shortly after surgery in our patient reflects a rectification of MG-associated anomalies in cellular and humoral immunity remains to be elucidated.

A previous study reported that patients with POMC had a worse prognosis compared with that in those without,^[30]^ highlighting the need for appropriate perioperative management for patients with MG to prevent the occurrence of POMC. In addition to avoiding the provoking factors (eg, β-blockers and nondepolarizing neuromuscular blocking agents),^[5]^ various anesthetic approaches have been reported to impact on postoperative outcomes in MG patients.^[31]^ For instance, patients receiving a combined anesthetic technique (ie, combined general and epidural analgesia) were found to have a lower incidence of postoperative ventilatory support than that in those undergoing balanced anesthesia after thymectomy.^[31]^ Several uncontrolled randomized clinical trials suggested that preoperative intravenous immunoglobulin may be beneficial to patients scheduled for thymectomy to prevent POMC,^32^,^33^ but this benefit was not found in another randomized control study.^[34]^

## 4 Conclusion

Myasthenic crisis and DVT are potential complications in patients with MG following thymectomy, underscoring the importance of avoiding intraoperative provoking factors and meticulous postoperative monitoring for early detection of myasthenic crisis to prevent life-threatening events as well as continual outpatient follow-up for possible thromboembolic complications. Hyperlactatemia may be a perioperative indicator of stress related to the triggering of myasthenic crisis.

## Author contributions

**Conceptualization:** Cheng-Yuan Lin, Min-Hsien Chiang, Jen-Yin Chen, Cheuk-Kwan Sun, Kuo-Chuan Hung.

**Data curation:** Wei-Cheng Liu, Wan-Jung Cheng.

**Formal analysis:** I-Ting Tsai, Shao-Chun Wu, Kuo-Chuan Hung.

**Investigation:** Jen-Yin Chen, Chin-Chen Chu, Cheuk-Kwan Sun.

**Methodology:** I-Ting Tsai, Shu-Wei Liao, Chin-Chen Chu.

**Writing – original draft:** Cheng-Yuan Lin, Wei-Cheng Liu, Cheuk-Kwan Sun, Kuo-Chuan Hung.

**Writing – review and editing:** Cheuk-Kwan Sun, Kuo-Chuan Hung.
